# Supplementary material for: Microbial effects of prebiotics, probiotics and synbiotics after Caesarean section or exposure to antibiotics in the first week of life: A systematic review
Source: PLoS One. 2022 Nov 9;17(11):e0277405. doi: 10.1371/journal.pone.0277405 (PMC9645654; doi:10.1371/journal.pone.0277405)
Supplement: Attachment — Submitted filename: Response to Reviewers.docx [file pone.0277405.s004.docx]

Amsterdam, October 2022

PLOS ONE

Dr. Tabatabaei-Malazy

Dear Dr. Tabatabaei-Malazy,

Thank you for the opportunity to revise our manuscript “Microbial effects of prebiotics, probiotics and synbiotics after Caesarean section or exposure to antibiotics in the first week of life: a systematic review”.

We thank the reviewers for taking the time to critically review and comment upon our manuscript. Our response to the reviewers’ comments can be found below. We hope this clarifies any ambiguities and that the revised manuscript meets the standards for publication in PLOS ONE.

We look forward to hearing from you regarding our submission, and we would be happy to address any further comments.

With kind regards,

on behalf of all authors,

Nora Carpay

**Reviewer #3**

Dear Authors,

The quality of the manuscript is improved. However, it should be considered following comments.

- Considering clinical trials as one of the inclusion criteria, why is not limited the search strategy to it? It could reduce the initial records, and time of assessment.

In hindsight, this would have been a good option. However, we wanted to make sure that we did not omit important studies of other study designs which we could include in our discussion. In the end, we did not find any relevant studies of other study designs.

- Please insert type of trials in table-1.

Thank you for your suggestion. To maintain the readability of table 1, we changed the title to “General characteristics of the included randomised controlled trials” instead of “General characteristics of the included studies” to clarify.

- According to following evidence, mode of delivery has influenced on gut microbiota of the infants. I suggest to exclude Zhong study and also revised the title and inclusion criteria.
  The mode of delivery affects the diversity and colonization pattern of the gut microbiota during the first year of infants' life: a systematic review. BMC Gastroenterol. 2016 Jul 30;16(1):86. doi: 10.1186/s12876-016-0498-0.

We agree that mode of delivery affects the gut microbiota of infants. However, so does the administration of antibiotics in the first week of life*. In this review we therefore aimed to investigate the effect of pre-, pro- and synbiotics on the gut microbiota of infants born via C-section or after antibiotic exposure. Unfortunately, we only found one study (by Zhong et al.) about the effects of such supplements on infants exposed to antibiotics, but since this was our original question, we do think it is important to include this study to illustrate the need for further research. Furthermore, we do not want to change our initial aim, as this would lead to reporting bias.

*Van Daele E, Kamphorst K, Vlieger AM, Hermes G, Milani C, Ventura M, Belzer C, Smidt H, van Elburg RM, Knol J. Effect of antibiotics in the first week of life on faecal microbiota development. Arch Dis Child Fetal Neonatal Ed. 2022 May 9:fetalneonatal-2021-322861.

**Reviewer #4**

Carpay and colleagues provided a summary of the research on the impact of prebiotics, probiotics, and symbiotics on the makeup of the intestinal microbiota in term infants who were delivered by Caesarean section or who had received antibiotics during the first week of life. The patient, group of patients, or issue being examined; the intervention; comparator interventions; and particular outcomes are the aspects that the authors have addressed that are essential for a systematic review. The rational of the systematic review appears reasonable for me and the authors synthesized the results in a very informative and efficient way. The purpose and backgrounds are appropriately addressed in the introduction and discussion section. Methods section was written at its best. Tables and figures were adequate and inform the readers all the necessary information. Whenever it is questioned, the authors offer the necessary clarification in the discussion area. The review's limitations as well as the ones of the included research have been discussed by the authors. The authors have assessed the potential for bias and taken into account probable RoBs. The manuscript as it is now acceptable for publication in my opinion.

We thank the reviewer for taking the time to review our manuscript, and for their positive feedback.
